# Understanding the Spectrum of Mild Clinical Outcomes and Novel Findings in Arterial Tortuosity Syndrome Among Qatari Patients: Implications of SLC2A10 Mutation

**DOI:** 10.3390/biomedicines13010159

**Published:** 2025-01-10

**Authors:** Muhammed Riyas K. Rahmath, Haytham Ibrahim, Muhammad Faiyaz-Ul-Haque, Zafar Nawaz, Ahmad Zitoun, Ahmed Hussein, Ahmed Sadek, Ayman El-Menyar, Reema Kamal, Hassan Al-Thani, Gulab Sher

**Affiliations:** 1 Pediatric Cardiology, Hamad General Hospital, Hamad Medical Corporation, Doha 3050, Qatar; 2 Pediatric Cardiology, Sidra Medicine, Doha 26999, Qatar; 3 Department of Pathology and Laboratory Medicine, Hamad Medical Corporation, Doha 3050, Qatar; 4 Department of Pediatric Laboratory Medicine, The Hospital for Sick Children, Toronto, ON M5G 1E8, Canada; 5 Department of Surgery, Hamad Medical Corporation & Weill Cornell Medicine, Doha 3050, Qatar; 6 Translational Research Institute, Academic Health System, Hamad Medical Corporation, Doha 3050, Qatar

**Keywords:** arterial tortuosity syndrome, SLC2A10, Qatari, founder mutation, mild outcome

## Abstract

**Background**/**Objectives**: Arterial Tortuosity Syndrome (ATS) is a rare, autosomal recessive connective tissue disorder characterized by arterial twists, abnormal bulges, constriction, and tears. Patients have distinctive features and disease manifestations. The syndrome’s full clinical spectrum and course remain incompletely understood. **Methods**: We sought to review the medical records of Qatari patients who had ATS. The cohort study included 21 patients who were genetically confirmed by mutations in the SLC2A10 gene. **Results**: The study revealed that the NM_030777.4(SLC2A10):c.243C>G (p.Ser81Arg) mutation in SLC2A10 leads to mild outcomes of no mortality and less morbidity. Novel features such as a flat philtrum, bulbous noses, bilateral nasolacrimal duct obstruction, allergic conjunctivitis, latent nystagmus, café au lait spots, eczema, dermatitis, allergic reactions, bilateral temporomandibular joint cysts, bilateral syndactyly (toes), parapelvic cysts, kidney malrotation, vesicoureteral reflux, and nephrolithiasis were identified in our cohort. Furthermore, rare features previously documented in a limited number of patients, including leg length discrepancy, epilepsy, and migraine headaches, were also observed in our cohort. **Conclusions**: Our data contributes new insights into the life course of ATS in Qatari patients. These findings underscore the importance of effective education strategies through repeated counseling aimed at preventing cousin marriage and the syndrome within the cohort.

## 1. Introduction

Arterial Tortuosity Syndrome (ATS) (MIM 208050) is a rare autosomal recessive connective tissue disorder, with less than 120 genetically verified cases in the literature [1,2,3,4,5,6]. It is characterized by a spectrum of anomalies affecting the vasculature, primarily involving the pulmonary arteries and branches of the aorta. These anomalies manifest as intricate twists, turns, and abnormal elongation of arteries, leading to tortuosity, constriction, and potential obstructions. The biomechanical consequence of arterial elongation, particularly in vessels with fixed endpoints, predisposes individuals to an increased risk of aneurysm formation, stenosis, and tears along the arterial intima, often resulting in arterial dissection and subsequent ischemia in affected organs [7,8,9,10,11,12,13]. Complications stemming from these arterial abnormalities can have severe clinical implications, including life-threatening scenarios such as massive hemorrhage from aneurysmal rupture or arterial dissection, as well as ischemic events culminating in strokes, respiratory distress, or myocardial infarction. While ATS can prove fatal in childhood, individuals with milder phenotypes may survive into adulthood [13,14].

Characteristic craniofacial features and connective tissue manifestations are commonly observed in individuals with ATS [7,10,11,12,13,15,16,17,18,19,20,21]. These include Marfanoid skeletal features, diaphragmatic hernia, cutis laxa, and hyperextensible skin. Despite significant advancements in understanding the syndrome, the complete clinical spectrum and natural course of ATS remain incompletely understood.

ATS is primarily attributed to mutations in the SLC2A10 gene, located on chromosome 20q13.2, which encodes the glucose transporter GLUT10. As understood so far, the main diagnostic criterion for the disease is SLC2A10. To date, 54 mutations have been cataloged in the SLC2A10 gene (Human Gene Mutation Database). While the precise role of GLUT10 remains under investigation, current evidence suggests its involvement in transporting dehydroascorbic acid, a vital hydroxylation cofactor necessary for prolyl and lysyl residues. The hydroxylation of these residues is critical for the maturation of elastin and collagen, and disruption due to loss-of-function mutations in GLUT10 contributes to the phenotypic expression of ATS [22]. Furthermore, GLUT10 deficiency alters the transforming growth factor β (TGF-β) pathway, disrupting various extracellular matrix proteins crucial for the structural integrity of blood vessel walls and other connective tissues [1].

Histological and ultrastructural analyses of skin and tissue biopsies from ATS patients have elucidated further insights into the pathogenesis of the syndrome. These studies have revealed fragmented elastic fibers, widened interstitial spaces between collagen fibers, and increased collagen deposition in both cutaneous and vascular tissues [2,23]. Reductions in elastin fiber quantity and alterations in texture, along with disorganization of connective tissue bundles, have been noted [2,24]. Additionally, skin arterioles in ATS patients exhibit absence of myofilaments, asymmetrical internal elastic lamina, and disorganized medial smooth muscle cells [23,25].

Despite significant progress, our current understanding of ATS remains limited, particularly in certain geographic regions such as Qatar, where only two studies have been conducted thus far [7,25]. Given the complexity of the syndrome and its variable clinical presentation, further research endeavors hold promise for unraveling additional insights into its pathophysiology and clinical management. In this study, we scrutinized the medical records of 21 Qatari ATS patients, shedding light on the specific genetic mutations and clinical outcomes associated with the syndrome in this population.

## 2. Materials and Methods

### 2.1. Ethical Approval

The study protocol received approval from the Institutional Review Board of HMC (HMC-IRB Registration: IRB-HMC-2021-011) on 26 September 2024.

### 2.2. Study Design

The study was envisioned as a retrospective descriptive analysis.

### 2.3. Study Population

In this cohort study, a comprehensive list of patients diagnosed with ATS was compiled from medical records. Patient medical records were meticulously reviewed to extract pertinent information related to ATS, with a particular focus on cardiovascular, craniofacial, and connective tissue manifestations. Based on the clinical manifestations of ATS, 67 patients had accessible medical records. These 67 patients were selected from the 01 January 1995 to 31 December 2018 time period. All the patients in the study belonged to Qatari ethnicity.

### 2.4. Exclusion Criteria

Out of the 67 patients, 45 patients did not have a genetic diagnosis; therefore, those patients were excluded. Furthermore, since another study [25] had reported the same mutation in August 2008 in Qatar, therefore one patient who had a date of birth of 15 June 2005 was also excluded.

### 2.5. Inclusion Criteria

Only 21 patients were selected in the study who were genetically confirmed by mutations in the SLC2A10 gene. 

### 2.6. Data Collection

Data collection took place at both Hamad Medical Corporation and Sidra Medicine, involving multidisciplinary teams comprising cardiologists, vascular surgeons, orthopedists, ophthalmologists, urologists, dermatologists, pulmonologists, gastroenterologists, neurologists, and clinical geneticists.

### 2.7. Data Analysis

Manual extraction of data from medical reports was performed to ensure accuracy and completeness. Subsequently, the collected data were organized in an Excel spreadsheet in charts to facilitate trend analysis and characterization of the clinical spectrum exhibited by the patients.

## 3. Results

### 3.1. Clinical Diagnosis

A total of 21 patients received a clinical diagnosis of ATS. Cardiologists conducted the diagnosis utilizing several imaging modalities, including echocardiography, CT angiography, and MRI, which were used on a case-by-case basis. The majority of patients exhibited significant tortuosity in the aorta, its branches, and pulmonary arteries. Additionally, facial dysmorphism was observed in a substantial proportion of patients, and various forms of hernias were prevalent among the cohort. Furthermore, skeletal abnormalities were identified in some patients.

### 3.2. Genetic Diagnosis

Genetic diagnosis was conducted by clinical geneticists, revealing pertinent findings in our patient cohort. Among them, twenty-one patients exhibited a homozygous pathogenic variant, NM_030777.4(SLC2A10):c.243C>G (p.Ser81Arg), in the SLC2A10 gene (Table S1). The variant is likely pathogenic according to ACMG classification as described below: (1) PM1: Located in a mutational hot spot and/or critical and well-established functional domain (e.g., active site of an enzyme) without benign variation: sugar transporter, major facilitator superfamily domain, (2) PM2: Absent from controls (or at extremely low frequency if recessive) in Exome Sequencing Project, 1000 Genomes Project, or Exome Aggregation Consortium, (3) PP3: Multiple lines of computational evidence support a deleterious effect on the gene or gene product (conservation, evolutionary, splicing impact, etc.), (4) PP5: Reputable source recently reports variant as pathogenic, but the evidence is not available to the laboratory to perform an independent evaluation (ClinVar: pathogenic/likely pathogenic). 

### 3.3. Demographic Features

The male-to-female (M/F) ratio in the cohort was 1.3:1, with 12 males and 9 females. The age range of patients in the cohort spanned from 19 months to 13 years, with a mean age of 5.05 years (std. deviation of 3.57), a median age of four years, and a range of 11.42. Fifteen patients were born from consanguineous marriages.

No mortality attributable to ATS was observed in this cohort. Family history played a significant role, with seven patients having affected relatives, primarily siblings. In one family, the patient’s father was also affected by ATS, while in another, the mother was affected. Additionally, one family opted for in vitro fertilization (IVF), resulting in twin daughters affected by ATS.

### 3.4. Craniofacial Manifestations

Craniofacial features were documented in twelve patients, encompassing a range of characteristics. These included facial dysmorphism (*n* = 12), a long, narrow face (*n* = 2), droopy cheeks (*n* = 7), blepharophimosis (*n* = 3), down-slanting palpebral fissures (*n* = 1), a high-arched palate (*n* = 3), micrognathia (*n* = 4), hypotelorism (*n* = 1), a beaked nose (*n* = 1), macrocephaly (*n* = 2), large ears (*n* = 1), low-set ears (*n* = 1), flat philtrum (*n* = 2), myopathic face (*n* = 1), flattened nasal bridge (*n* = 1), bulbous nose (*n* = 2), and telecanthus (*n* = 1).

### 3.5. Cutaneous Manifestations

Cutaneous findings were noted in twelve patients, encompassing hyperextensible skin (*n* = 8), eczema (*n* = 1), dermatitis (*n* = 2), thin skin (*n* = 1), allergic skin rash (*n* = 1), velvety texture (*n* = 2), allergy to various foods (*n* = 1), and café au lait spots in one patient.

### 3.6. Atopic Features

Five patients exhibited atopic features, including eczema (*n* = 1), dermatitis (*n* = 2), and allergic reactions (*n* = 2).

### 3.7. Ocular Anomalies

Seven patients presented with ocular anomalies, including astigmatism (*n* = 5), myopia (*n* = 2), bilateral nasolacrimal duct obstruction (*n* = 1), allergic conjunctivitis (*n* = 2), microphthalmia (*n* = 1), and latent nystagmus (*n* = 1).

### 3.8. Skeletal Abnormalities

Skeletal abnormalities were observed in fifteen patients, including hypermobile joints (*n* = 8), scoliosis (*n* = 2), long, thin fingers (*n* = 2), limited extension of the right elbow (*n* = 1), pectus excavatum (*n* = 3), bilateral talipes equinovarus (*n* = 1), bilateral flat foot, in-toeing, and planovalgus (*n* = 2), a leg length discrepancy (*n* = 1), bilateral temporomandibular joint cyst (*n* = 1), muscular hypotonia (*n* = 8), joint contractures (*n* = 1), muscle weakness (*n* = 1), tending to slouch forward (*n* = 1), hips Internal rotation (*n* = 1), claw hand (*n* = 1), and bilateral syndactyly (*n* = 1).

### 3.9. Respiratory Manifestations

Thirteen patients exhibited respiratory manifestations, including recurrent chest infections (*n* = 8), diaphragmatic eventration (*n* = 1), laryngomalacia (*n* = 1), reactive airway disease (*n* = 1), pneumonia (*n* = 1), obstructive sleep apnea (*n* = 1), cystic lesions (*n* = 1), tachypnea (*n* = 2), and asthma (*n* = 1). Additionally, five patients experienced respiratory distress syndrome.

### 3.10. Hernias

Inguinal hernias were reported in eight patients, with one experiencing recurrent occurrences. Two patients had umbilical hernias, while diaphragmatic hernias were noted in seven patients, including two with Morgagni’s hernia. Hiatal hernias were found in five patients. Additional findings included bilateral diaphragmatic eventration, lung herniation into the neck, and subglottic narrowing in a patient.

### 3.11. Urogenital Anomalies

Bladder diverticula were present in two patients. Other findings included a small right renal parapelvic cyst and abnormal left kidney location with malrotation in one patient each. Vesicoureteral reflux, bilateral nephrolithiasis, debris in the bladder, a dilated pyelocaliceal system, and left proximal hydroureter were noted in one patient each.

### 3.12. Neurological Anomalies

One patient had epilepsy, while four experienced migraine headaches. Subgaleal hematoma was documented in one patient. Other anomalies include cephalohematoma (*n* = 1), photophobia (*n* = 1), phonophobia (*n* = 1), speech delay (*n* = 2), behavioral difficulties (*n* = 1), developmental delay (*n* = 1), and head lag (*n* = 1).

### 3.13. Gastroesophageal Manifestations

Eleven patients exhibited gastroesophageal manifestations, including gastroesophageal reflux (*n* = 5), abdominal pain (*n* = 1), chronic constipation (*n* = 2), FTT (*n* = 3), feeding difficulties (*n* = 3), bowel obstruction (*n* = 1), pyloric stenosis (*n* = 1), aversion to particular food (*n* = 1), and rectal prolapse (*n* = 1).

### 3.14. Cardiovascular Manifestations

**Figure 1:**
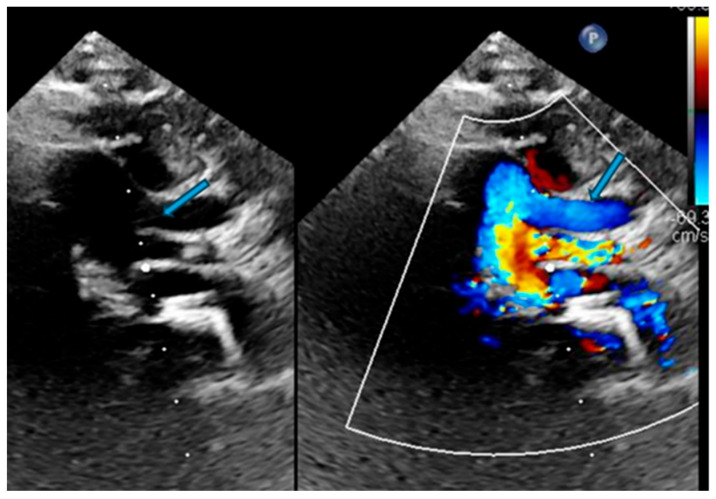
Echocardiogram image showing tortuous aortic arch branches (blue arrow) in a patient.

**Figure 2:**
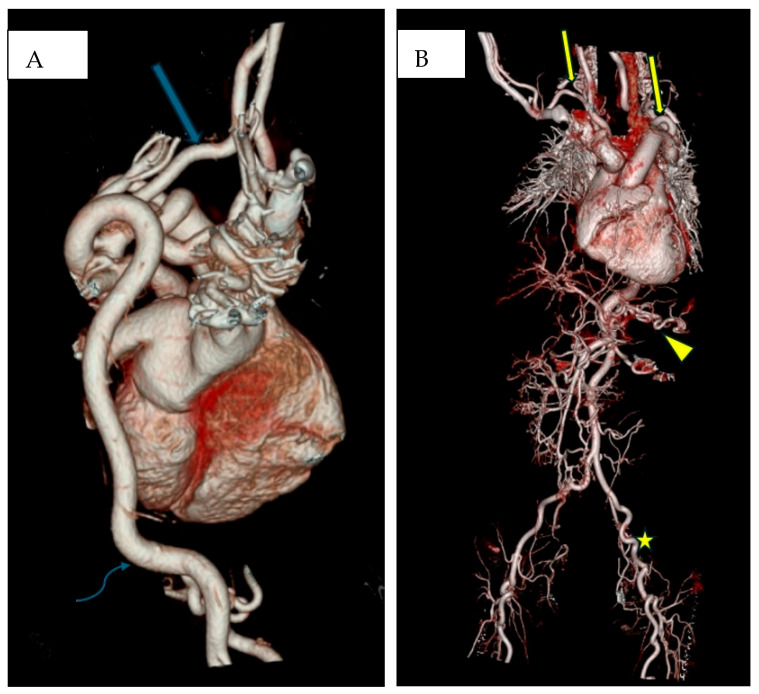
(**A**): Three-dimensional (3D) rendering from CT angiography shows tortuosity of the aortic arch and its branches (blue arrows) in a patient. (**B**): 3D rendering from CT angiography shows tortuosity of the aortic arch and its branches (yellow arrow), an elongated abdominal aorta with its coiled branches (yellow arrowhead), along with tortuosity of the lower limb arteries (asterisk) in a patient.

Tortuosity of the aorta was present in 21/21 (100%) patients, while tortuosity of other arteries was seen in 20/21 (95%) patients. Tortuosity in other arteries included pulmonary arteries (19/21 = 90%), common carotid arteries (2/21 = 9%), head and neck vessels (3/21 = 14%), radial (1/21 = 4%), ulnar (1/21 = 4%), renal (1/21 = 4%), subclavian (1/21 = 4%), iliac (1/21 = 4%), brachiocephalic (1/21 = 4%), coronaries (1/21 = 4%), and aorta arch branches (Figure 1 and Figure 2) (1/21 = 4%).

Other cardiovascular abnormalities include aortic root aneurysm (2/21 = 9%), abnormal implantation of the aortic branches (1/21 = 4%), early and abnormal branching of the pulmonary arteries (1/21 = 4%), right atrial dilatation (2/21 = 9%), medium sized secundum ASD (2/21 = 9%), patent ductus arteriosus (2/21 = 9%), mild cardiac displacement to the right (1/21 = 4%), mild interventricular septal hypertrophy (1/21 = 4%), stenosis of the pulmonary arteries (4/21 = 19%), stenosis in ascending aorta (1/21 = 4%), tortuous inferior vena cava (2/21 = 9%), PFO shunting left to right (4/21 = 19%), mild tricuspid valve regurgitation (1/21 = 4%), mild prolapse of posterior mitral valve leaflet (1/21 = 4%), and left ventricular hypertrophy (1/21 = 4%).

Surgical interventions were required for 2/21 (9%) patients, including balloon dilatation in the right femoral artery and cardiovascular therapeutic catheterization in one patient, while multiple balloon dilatations of pulmonary artery branch stenosis were performed in another patient.

## 4. Discussion

ATS is a rare genetic disease with heterogeneous phenotypic features as per reports [1]. There are two studies that have been conducted in Qatar. Study-1 described the clinical spectrum of manifestations in a cohort of 32 patients [7], while study-2 identified a homozygous p.Ser81Arg mutation in the SLC2A10 gene in 14 patients [25]. The mutation likely shares a common origin in Middle Eastern patients, as it has been described in Middle Eastern families [24]. In our study, we described a cohort of 21 Qatari patients with a clinical and genetic diagnosis of ATS. These 21 patients carried the homozygous p.Ser81Arg mutation in the SLC2A10 gene. Since the study-2 mentioned above identified the p.Ser81Arg in August 2008, therefore the 21 patients in this study who have genetic diagnosis and birth date after 2008 are new patients and do not include any candidates from the previous study.

Initial studies reported that ATS is fatal in infancy [13]; however, later studies show a milder phenotype [14]. There is no mortality and less morbidity in our cohort, and all patients are hemodynamically stable. Only 2/21 (9%) patients required surgical intervention for cardiovascular manifestations, which was successful. Ten out of the 21 patients (47%) required surgeries for non-cardiovascular manifestations, mostly hernias and rarely other connective tissue anomalies. There was no aneurysmal dilation of the intracranial arteries. Most of the patients are children, for whom the diseases have been described as fatal in the literature. Our findings suggest that the outcome of the mutation in Qatari patients seems to be mild. Although the patients in our cohort are stable, their quality of life has been affected due to frequent hospital visits, multiple surgeries, and multiple respiratory manifestations and skeletal anomalies.

There was no difference in sexual predilection of ATS observed in our study as reported before [2]. However, the extra-cardiovascular manifestations, including craniofacial dysmorphism, ocular, cutaneous, musculoskeletal, hernia, gastrointestinal, urogenital, and respiratory manifestations, were not universally documented in medical records. Consequently, their prevalence in Table 1 may lack reliability. The prevalence of respiratory manifestations in our cohort (13/21 = 61%) surpasses that reported in the literature (10/67 = 15%) [2] however the respiratory manifestations in our cohort may be considered more specific for p.Ser81Arg variant while that reported in the literature specific for a mix of variants. Although cardiovascular data were comprehensively collected for all patients and largely conformed to published data [2], instances of pulmonary artery stenosis in our cohort (19%) were fewer compared to the reported 57% in the literature [2].

Previous studies have not established a genotype-phenotype correlation in ATS [1], a trend we also observed in our cohort. Interestingly, our study identified numerous manifestations not previously described in the literature, shedding light on the life course of Qatari patients with ATS. For instance, two patients exhibited a flat philtrum instead of the long philtrum commonly noted [2], while one other displayed a bulbous nose contrary to the described beaked nose [2]. Cutaneous manifestations like café au lait spots were observed in one patient, warranting further investigation. Five patients presented with atopic features, possibly linked to increased immune signaling resulting from loss of function mutation in the SLC2A10 gene.

In a recent study on ATS, the role of GLUT10, encoded by the SLC2A10 gene, in immunity was investigated [26]. The study revealed that knocking down SLC2A10 led to a widespread increase in immune and inflammatory signaling, with a notable dependence on SLC2A10 expression for the infiltration of various immune cells, particularly macrophages. The authors hypothesized that SLC2A10 might regulate this infiltration via the COX-2 pathway. In our cohort, loss-of-function mutations in SLC2A10 potentially heightened immune signaling, resulting in atopic anomalies.

Remarkably, we observed ocular anomalies such as bilateral nasolacrimal duct obstruction, allergic conjunctivitis, and latent nystagmus, which have not been previously described in the literature in association with ATS. Similarly, skeletal abnormalities, including bilateral TMJ cysts and bilateral syndactyly of the toes, were noted, which are uncommon in the ATS spectrum. Additionally, we identified a rare occurrence of leg length discrepancy, expanding the understanding of ATS manifestations.

Furthermore, our cohort exhibited urogenital manifestations consistent with previous literature findings. However, we identified novel findings such as parapelvic cysts, kidney malrotation, vesicoureteral reflux, and nephrolithiasis, which warrant further investigation. Neurological anomalies, notably epilepsy and migraine headaches, were more prevalent in our cohort compared to previous reports. Subgaleal hematomas were also observed, aligning with recent mentions in ATS cases.

ATS follows an autosomal recessive inheritance pattern, with implications for familial recurrence. We emphasize scenarios where both parents are heterozygous or one parent is affected and the other is a carrier, significantly impacting the odds of having an afflicted offspring. Notably, in one instance, despite the risk, a couple opted for in vitro fertilization (IVF), resulting in dizygotic twins, both affected by ATS. This highlights the potential benefits of preimplantation genetic testing to prevent inherited disorders.

The clinical spectrum of ATS is broad, ranging from infancy to adulthood, with varying severity. Aneurysm formation and dissection pose significant risks at any age, emphasizing the need for continuous monitoring and early intervention. While mortality has been absent thus far, vigilance is necessary to detect and manage potential cardiovascular complications promptly.

One of the study’s major limitations is that not all medical records contain all the information. The study’s extremely high percentage of children (15 out of 21) from consanguineous marriages is another drawback. Because of this, some of the clinical symptoms in our group might be linked to consanguineous marriages and homozygosity at other loci. Therefore, many children from consanguineous marriages may have symptoms that are not typical of ATS. Furthermore, some of the features (eczema, dermatitis, allergic reactions) may be quite frequent in the normal population and may not necessarily be related to the syndrome.

## 5. Conclusions

We have presented a cohort of 21 patients with ATS, who have received a genetic diagnosis through the detection of mutations in SCL2A10. Our data contributes new insights into the life course of ATS in Qatari patients. While the outcome of ATS in Qatari patients appears to be mild, the presence of comorbidities has significantly impacted the quality of life in these individuals. Addressing the strong cultural barrier of cousin marriage through effective educational strategies may help prevent the syndrome in future generations.

## Figures and Tables

**Table 1:** Phenotypic manifestation of ATS in the Qatari cohort.

Feature	Prevalence
M/F ratio	12/9 (1.3:1)
**Parental consanguinity**	**15/21 (71%)**
Family history of ATS	7/21 (33%)
**Craniofacial dysmorphism**	**12/21 (57%)**
Ocular manifestations	7/21 (33%)
**Cutaneous manifestations**	**12/21 (57%)**
**Musculoskeletal manifestations**	**15/21 (71%)**
Diaphragmatic hernia	7/21 (33%)
Inguinal hernia	8/21 (38%)
Umbilical hernia	2/21 (9%)
Hiatal hernia	5/21 (23%)
**Gastrointestinal manifestations**	**11/21 (52%)**
Urogenital manifestations	5/21 (23%)
**Respiratory manifestations**	**13/21 (61%)**
Non-cardiovascular surgery	10/21 (47%)
**Aortic tortuosity**	**21/21 (100%)**
**Tortuosity of other arteries**	**20/21 (95%)**
Abnormal implantation of the aortic branches	1/21 (4%)
Aortic root aneurysm	2/21 (9%)
Other arterial aneurysms	0/21 (0%)
Arterial dissections	0/21 (0%)
Stenosis of the pulmonary arteries	4/21 (19%)
Aortic stenosis	1/21 (4%)
Other arterial stenoses	0/21 (0%)
Surgical intervention	2/21 (9%)
**Mutation in SLC2A10**	**21/21 (100%)**

Features in which the prevalence is >50% are in bold letters.

## Data Availability

The data in this study is available in ClinVar with accession number VCV000004588.23.

## Supplementary Materials

The following supporting information can be downloaded at https://www.mdpi.com/article/10.3390/biomedicines13010159/s1, Table S1: Genotypes and the ACMG classification of ATS patients. References [23,24,25] have been cited in the supplementary materials.
