# Supplementary material for: Understanding the Spectrum of Mild Clinical Outcomes and Novel Findings in Arterial Tortuosity Syndrome Among Qatari Patients: Implications of SLC2A10 Mutation
Source: Biomedicines. 2025 Jan 10;13(1):159. doi: 10.3390/biomedicines13010159 (PMC11763099; doi:10.3390/biomedicines13010159)
Supplement: Supplementary file 1 [file biomedicines-13-00159-s001.zip › biomedicines-3369665-supplementary.pdf]

Genetic diagnosis was conducted by clinical geneticists. Depending on the availability of details about the mutation in SLC2A10 in medical records, only 10 patients have an ACMG-classified likely pathogenic mutation.

Table S1. Genotypes and the ACMG classification of ATS patients.

Patient ID	Mutation in SLC2A10 (NM_030777.4)	Zygosity	ACMG classification	Previously published in
ATS01	Positive: c.243C>G (p.Ser81Arg)	Homozygous	Likely Pathogenic	[23,24,25]
ATS02	Positive: c.243C>G (p.Ser81Arg)	Homozygous	Likely Pathogenic	[23,24,25]
ATS03	Positive: c.243C>G (p.Ser81Arg)	Homozygous	Likely Pathogenic	[23,24,25]
ATS04	Positive: c.243C>G (p.Ser81Arg)	Homozygous	Likely Pathogenic	[23,24,25]
ATS05	Positive: c.243C>G (p.Ser81Arg)	Homozygous	Likely Pathogenic	[23,24,25]
ATS06	Positive*	Homozygous	-	
ATS07	Positive*	Homozygous	-	
ATS08	Positive*	-	-	
ATS09	Positive*	-	-	
ATS10	Positive*	-	-	
ATS11	Positive*	-	-	
ATS12	Positive*	Homozygous	-	
ATS13	Positive: c.243C>G (p.Ser81Arg)	Homozygous	Likely Pathogenic	[23,24,25]
ATS14	Positive*	Homozygous	-	
ATS15	Positive: c.243C>G (p.Ser81Arg)	Homozygous	Likely Pathogenic	[23,24,25]
ATS16	Positive: c.243C>G (p.Ser81Arg)	Homozygous	Likely Pathogenic	[23,24,25]
ATS17	Positive: c.243C>G (p.Ser81Arg)	Homozygous	Likely Pathogenic	[23,24,25]
ATS18	Positive*	Homozygous	-	
ATS19	Positive*	Homozygous	-	
ATS20	Positive: c.243C>G (p.Ser81Arg)	Homozygous	Likely Pathogenic	[23, 24, 25]
ATS21	Positive*	Homozygous	-	

* Detail unknown
